# Diffusion Restriction in the Splenium: A Comparative Study of Cytotoxic Lesions of the Corpus Callosum (CLOCCs) versus Lesions of Vascular Etiology

**DOI:** 10.3390/jcm12226979

**Published:** 2023-11-08

**Authors:** Brian Stamm, Christina M. Lineback, Mengxuan Tang, Dan Tong Jia, Ella Chrenka, Farzaneh A. Sorond, Behnam Sabayan

**Affiliations:** 1 Department of Neurology, Feinberg School of Medicine, Northwestern University, Chicago, IL 60611, USA; 2 Department of Neurology, University of Michigan, Ann Arbor, MI 48109, USA; 3 Department of Neurology, University of Minnesota Medical School, Minneapolis, MN 55415, USA; 4 Healthpartners Institute, Healthpartners, Bloomington, MN 55425, USA; 5 Hennepin Healthcare Research Institute, Department of Neurology, Hennepin County Medical Center, Minneapolis, MN 55404, USA; 6 Division of Epidemiology and Community Health, School of Public Health, University of Minnesota, Minneapolis, MN 55455, USA

**Keywords:** splenium, diffusion restriction, cytotoxic lesion of the corpus callosum, ischemic stroke

## Abstract

Cytotoxic lesions of the corpus callosum (CLOCCs) have broad differential diagnoses. Differentiating these lesions from lesions of vascular etiology is of high clinical significance. We compared the clinical and radiological characteristics and outcomes between vascular splenial lesions and CLOCCs in a retrospective cohort study. We examined the clinical and radiologic characteristics and outcomes in 155 patients with diffusion restriction in the splenium of the corpus callosum. Patients with lesions attributed to a vascular etiology (N = 124) were older (64.1 vs. 34.6 years old, *p* < 0.001) and had >1 vascular risk factor (91.1% vs. 45.2%, *p* < 0.001), higher LDL and A1c levels, and echocardiographic abnormalities (all *p* ≤ 0.05). CLOCCs (N = 31) more commonly had midline splenial involvement (*p* < 0.001) with only splenial diffusion restriction (*p* < 0.001), whereas vascular etiology lesions were more likely to have multifocal areas of diffusion restriction (*p* = 0.002). The rate of in-hospital mortality was significantly higher in patients with vascular etiology lesions (*p* = 0.04). Across vascular etiology lesions, cardio-embolism was the most frequent stroke mechanism (29.8%). Our study shows that corpus callosum diffusion restricted lesions of vascular etiology and CLOCCs are associated with different baseline, clinical, and radiological characteristics and outcomes. Accurately differentiating these lesions is important for appropriate treatment and secondary prevention.

## 1. Introduction

The corpus callosum (CC) is the brain’s largest white matter tract and the major commissural pathway connecting and allowing crosstalk between the two hemispheres. It also affords one hemisphere control and inhibition of the corresponding areas from the other hemisphere, which leads to hemispheric specialization of brain function [1]. Due to these critical processes, accurate and timely detection of CC lesions both clinically and radiologically is imperative, particularly when a vascular etiology is present that could be intervened on.

Although it is always crucial to consider ischemic infarction in the differential diagnosis of diffusion restriction, splenial diffusion restricting lesions, in particular, have a multitude of potential etiologies [2]. For example, the characteristic morphology of a cytotoxic lesion of the corpus callosum (CLOCC)—midline, symmetric, and usually splenial—invokes a wide differential, including exposures to neuro-active medications (e.g., metronidazole), trauma, toxic-metabolic abnormalities, subarachnoid hemorrhage, infection (e.g., viral encephalitis), and malignancy, among others [3]. The splenium in particular may be implicated given its abundance of neurotransmitter receptors, making it selectively vulnerable to cytokinopathy and cytotoxic edema [3]. Infarcts may also occur in the CC but it is reported to be a rare phenomenon, accounting for 3–8% of infarcts at large [4]. The rarity of callosal infarction is posited to be secondary to the abundant collateral blood supply of the CC from both the anterior and posterior circulation [5,6]. Although perhaps rare at large, in a recent study we found that among two hundred splenial diffusion restricted lesions, vascular etiologies were most common [7]. This study also systematically reported outcomes from splenial diffusion restricting lesions, showing that in-hospital mortality occurred in 8.5% of cases, and nearly half were readmitted to the hospital within 1 year [7]. To our knowledge, only one prior study has compared the imaging and clinical characteristics between vascular and non-vascular etiologies of diffusion restriction in the splenium of the CC [2], and no prior studies have directly compared vascular lesions with CLOCCs. Accurate differentiation between these distinct etiologies of CC lesions is important given that it influences both the acute and long-term management of these patients.

More precise determination of the radiological and clinical characteristics and outcomes of lesions of vascular etiology versus CLOCCs would optimize the acute treatment and secondary prevention of the vascular etiology lesions, while helping to differentiate them from other distinct etiologies which require specific management. We performed a large retrospective chart review to compare the clinical and radiological characteristics and outcomes between vascular and CLOCC etiologies of splenial diffusion restricted lesions.

## 2. Materials and Methods

### 2.1. Study Population

Our methods detailing the creation of the retrospective database for this study have been previously published [7]. Briefly, we searched the clinical radiology database at a single, tertiary academic institution for cases of diffusion restriction within the splenium of the corpus callosum in patients aged ≥ 18 over the time period from 1 January 2009–1 August 2020. We attempted to minimize selection bias by starting with an inclusive and comprehensive search strategy, subsequently narrowing the patient sample by a systematic review of the imaging [7]. Diffusion restriction was defined by increased (bright) signal on diffusion weighted imaging (DWI) and decreased (dark) signal on apparent diffusion coefficient (ADC). Patients with prior neurosurgical procedures, recent cardiac procedures, hemorrhage-associated diffusion restriction, diffuse anoxic brain injury, chronic or previously characterized disease processes, and those without a defined CLOCC or vascular etiology for the splenial diffusion restriction were excluded. The rationale for these exclusion criteria was to ensure that the study sample consisted primarily of undifferentiated cases of splenial diffusion restriction to match the cases encountered in clinical practice. The etiology for the splenial diffusion restriction in each case was documented by the treating medical team. Thus, vascular etiology lesions were defined as those in which the medical team documented a cerebrovascular process, e.g., anterior cerebral artery infarction affecting the splenium, as the most likely etiology for the patient’s splenial diffusion restriction. Additionally, for vascular etiology lesions, the most likely stroke etiology was recorded per TOAST criteria [8], as determined by the treating medical team’s electronic medical record notes. CLOCCs were defined by the characteristics previously published by Starkey et al.: (1) a small round lesion in the center of the splenium, (2) a lesion centered in the splenium, extending laterally through the nearby callosal fibers, or (3) a lesion located in the posterior splenium extending anteriorly [3]. All MRI neuroimaging was performed either using 1.5- or 3-Tesla scanners, and all imaging had formal neuroradiology reports. All neuroimages and radiology reports were reviewed during the chart abstraction phase. There were systematic quality control checks in place at multiple points throughout the chart abstraction process, as previously described [7]. The study was approved by our Institutional Review Board (study number STU00210797).

### 2.2. Outcomes

The primary outcomes were readmissions within 1 year, resolution of diffusion restriction within 1 year, and in-hospital mortality. Secondary outcomes included specific medication classes prescribed on discharge from the hospitalization, including antiplatelets, anticoagulation, and statins.

### 2.3. Covariates and Additional Variables

The following variables were included in the chart abstraction for all cases: demographic variables (age, gender, race, and ethnicity), medical history and vascular risk factors (hypertension, hyperlipidemia, obesity, diabetes, tobacco use, history of stroke or TIA, atrial fibrillation, valvular heart disease, myocardial infarction, coronary artery disease, systolic heart failure, family history of stroke or TIA, malignancy), and medications on admission (antiplatelets, anticoagulation, statins, antidepressants, antiepileptic drugs, antipsychotics, chemotherapies, and antibiotics). Clinical characteristics (including lab values such as low-density lipoprotein (LDL) and hemoglobin A1c, electrocardiogram (EKG) findings, and echocardiographic findings) and radiological data (including regions of corpus callosal diffusion restriction, patterns of splenial involvement, other areas of diffusion restriction, and other radiological findings) were also collected.

### 2.4. Statistical Analyses

Descriptive statistics were generated for the baseline characteristics (demographic, medical history and vascular risk factors, and medications on admission), clinical and radiological characteristics of patients with vascular and CLOCC etiologies of splenial diffusion restriction. Comparisons were performed between the characteristics of the vascular etiology lesions and CLOCCs using Fisher’s Exact test due to small counts in the CLOCC group. The only exception was that a t-test with equal variances was used for comparison of age between the groups. The frequency of outcomes included the 95% confidence intervals for each of the proportions, and Fisher’s Exact Test was used to compare proportions (appropriate for small counts from CLOCC group). Outcomes were then compared using unadjusted and adjusted logistic regression. Covariates for adjustment included baseline patient characteristics age, sex, race, and ethnicity. If a patient was missing a variable, it was treated as the absence of the respective variable.

## 3. Results

**Figure 1:**
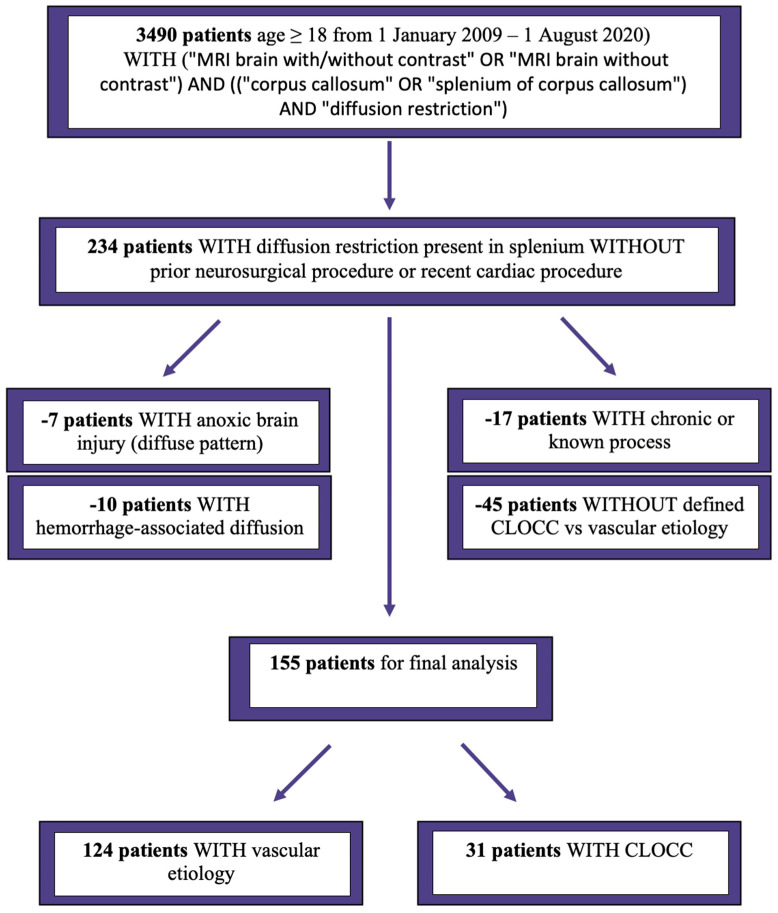
Flowsheet depicting the initial search inclusion criteria and the application of exclusion criteria to yield the study population.

After inclusion and exclusion criteria were applied, there were 124 patients with a vascular etiology of splenial diffusion restriction and 31 patients with CLOCCs (Figure 1). Patients with vascular etiology lesions were older than those with CLOCCs (64.1 vs. 34.6, *p* < 0.001), were less commonly Hispanic or Latino (8.9% vs. 25.8%, *p* = 0.02), more frequently had >1 vascular risk factor (91.1% vs. 45.2%, *p* < 0.001), were taking antiplatelet (*p* < 0.001) and statin medications (*p* < 0.001) on admission (Table 1), had elevated LDL (*p* = 0.027) and A1c (*p* < 0.001), and had echocardiographic abnormalities (*p* < 0.001). Patients with CLOCCs more commonly had midline splenial involvement (*p* < 0.001) with only the splenium affected (*p* < 0.001), whereas those with vascular etiology lesions had more multifocal (*p* = 0.002), cortical (*p* < 0.001), and other subcortical (*p* < 0.001) areas of diffusion restriction (Table 2).

The rate of in-hospital mortality was significantly higher in patients with vascular etiology lesions (*p* = 0.04) compared to CLOCCs. There were no statistically significant differences between the vascular etiology lesions and CLOCCs with respect to readmission within 1 year or resolution of diffusion restriction within 1 year. In the unadjusted analyses, odds of antiplatelet on discharge were 92% lower in those with CLOCCs as compared to patients with vascular etiology lesions (OR 0.08, 95% CI 0.02–0.21, *p* < 0.001) and 85% lower in the adjusted model (OR 0.15, 95% CI 0.04–0.49, *p* = 0.003). Odds of anticoagulant on discharge were 80% lower in CLOCCs compared to vascular etiology lesions in the unadjusted analysis (OR 0.20, 95% CI 0.03–0.71, *p* = 0.03), but this became nonsignificant in the adjusted analysis. Finally, the odds of statin on discharge were 96% lower in CLOCCs compared to vascular etiology lesions in the unadjusted analysis (OR 0.04, 95% CI 0.01–0.13, *p* < 0.001) and 88% lower in the adjusted analysis (OR 0.12, 95% CI 0.02–0.54, *p* = 0.01; Table 3).

**Figure 2:**
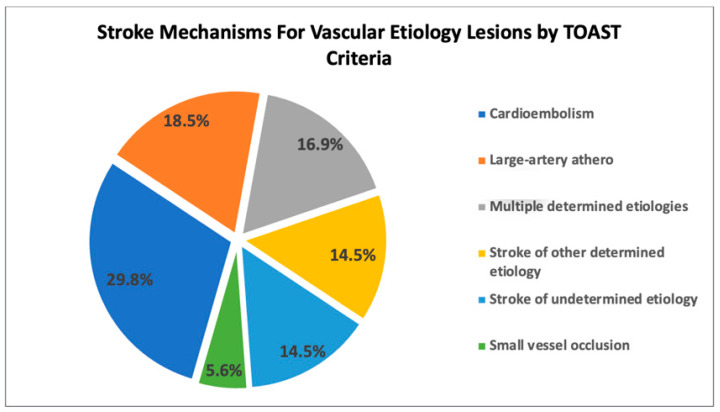

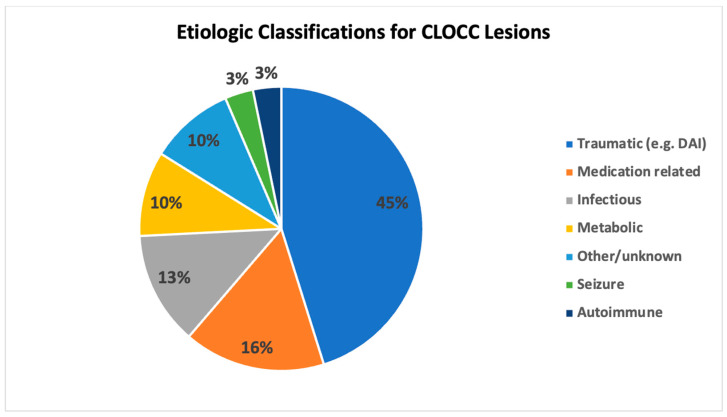
Breakdown of stroke mechanisms for vascular etiology lesions and etiologic classifications for CLOCC lesions.

Finally, as briefly reported in our prior study [7], the vascular stroke mechanisms included: cardioembolism (29.8%), large-artery atherosclerosis (18.5%), multiple determined etiologies (16.9%), stroke of other determined etiology (14.5%), stroke of undetermined etiology (14.5%), and small vessel occlusion (5.6%; Figure 2). CLOCCs were most frequently due to trauma (e.g., diffuse axonal injury), followed by medication-related, infectious, metabolic, other/unknown, seizure, and autoimmune etiologies for the splenial diffusion restriction (Figure 2).

## 4. Discussion

In this large retrospective study, patients with splenial vascular etiology lesions and CLOCCs had distinct demographic, clinical, and radiological characteristics, and outcomes. Patients with vascular etiology lesions had more vascular risk factors and echocardiographic abnormalities, and they were more likely to have multifocal areas of diffusion restriction. Patients with CLOCCs more commonly had midline splenial diffusion restriction with only the splenium affected. As for outcomes, patients with vascular etiology lesions were more likely to develop in-hospital mortality. Additionally, amongst cases with vascular etiology lesions, a cardioembolic stroke mechanism was most common. Accurate differentiation of vascular and CLOCC etiologies of splenial diffusion restriction is vital for appropriate acute management and secondary stroke prevention.

A prior study by Wilson et al. examined characteristics of vascular versus non-vascular etiologies for CC diffusion restriction, and, similar to our findings, showed that nonvascular cases were younger and had less vascular risk factors [2]. Whereas this prior study examined diffusion restricted lesions throughout the entire corpus callosum, the focus of our current study was the splenium, given its unique susceptibility to cytokinopathy and cytotoxic edema secondary to its exceedingly high density of neurotransmitter receptors [3,9,10]. This selective vulnerability predisposes the splenium to diffusion restriction on MRI imaging from a broad array of etiologies, such as those seen in CLOCCs [3]. While ischemic infarction is overall rare in the CC [4], the splenium is the most commonly affected region, occurring in 63% of vascular cases in the large retrospective study by Wilson et al. [2]. The splenium’s propensity for diffusion restriction resulting from either cytotoxic or infarct-mediated pathways may lead to uncertainty when clinicians encounter such lesions and must decide which etiology is more likely. Our findings suggest there are several baseline, clinical, and radiological characteristics that can be used to differentiate these lesions clinically. For instance, given that vascular lesions more frequently had lateralized splenial and multifocal areas of diffusion restriction, when clinicians encounter such radiographic characteristics, it may prompt consideration of a vascular etiology with appropriate diagnostic workup.

We also show that vascular and CLOCC etiologies for splenial diffusion restriction have distinct clinical outcomes. The higher in-hospital mortality related to vascular etiology lesions in our study has clinical plausibility, given that patients with vascular etiology lesions were older—though the adjusted models accounted for age—and had higher frequencies of vascular risk factors, such as diabetes, which have previously been shown to be associated with in-hospital mortality in a variety of medical conditions [11,12]. Prior literature on clinical outcomes from midline splenial lesions has generally been favorable [13], though prior studies are largely limited to case reports [14,15] and series [16]. The higher in-patient mortality for those with vascular etiology lesions may help inform prognostication. We hypothesized that CLOCCs would have been more likely to have reversal of diffusion restriction on neuroimaging, given that this is considered a hallmark finding of these lesions [17]. However, given the retrospective nature of the study, follow-up imaging was not standardized in either the vascular or CLOCC cohorts, which may have limited our ability to detect such a difference. Additionally, in ischemic infarction, the high signal intensity on diffusion-weighted imaging tends to normalize at 10–14 days after the ictus [18], and higher rates of reversible diffusion restriction findings have been seen in patients treated with thrombolysis [19].

Our finding that cardio-embolism was the most frequent stroke mechanism in vascular cases of splenial diffusion restriction is largely consistent with findings from prior series suggesting a higher rate of embolism in the posterior CC, whereas large artery atherosclerosis tends to be a more common etiology in the anterior CC [20,21]. The prior large, retrospective study by Wilson et al. found that atypical causes of infarct—such as vasculopathy/vasculitis and hypercoagulability—were the most common etiology for infarction of the CC, especially in cases with splenial involvement. Cardioembolism was the second most common etiology in their cohort, and similar to our study, small vessel disease was uncommon [2]. Their study—similarly to ours—was conducted at a single, academic institution, and case population mixes may vary, thus accounting for some of the small differences in our samples. Importantly, our findings collectively suggest that clinicians should consider cardioembolism and other more atypical causes of stroke when encountering diffusion-restricted lesions in the splenium.

Our study had several limitations. First, there were missing data for follow-up imaging and several other variables of interest. Because missing data were treated as the absence of the respective variable, we could be undercounting the true proportion of patients with certain outcomes. This may bias our results if the probability of missing data is systematically associated with either vascular etiology lesions or CLOCCs. While this limitation could be overcome by prospective studies on splenial lesions, such studies are relatively unlikely to occur given the infrequency of presentation for these lesions. Second, the adjusted models included demographic covariates but no covariates reflecting illness severity, as there was no standardized severity marker available from chart abstraction given the heterogeneity of pathophysiology within the study population. Third, because of our focus on diffusion restriction in the splenium of the CC, our findings regarding the vascular etiologies may not generalize to all types of callosal infarcts, which can also affect other regions of the CC [2]. Finally, this study was conducted at a single, academic tertiary care facility, and future studies could consider multicenter involvement to enhance generalizability.

## 5. Conclusions

CC diffusion restricted lesions of vascular etiology and CLOCCs are associated with different baseline, clinical, and radiological characteristics and outcomes. Clinically, patients with vascular lesions had a greater burden of vascular risk factors. Radiographically, vascular lesions more commonly had lateralized splenial and multifocal other areas of diffusion restriction. These features may be helpful to clinicians when ascertaining etiology for these lesions. Accurately differentiating these lesions is important for appropriate treatment and secondary prevention.

## Figures and Tables

**Table 1:** Baseline characteristics of patients with vascular and CLOCC etiologies of diffusion restriction in the splenium of the corpus callosum.

	Vascular	CLOCC	*p* Value
	N (%)	N (%)	
Demographics			
Number of patients	124	31	n/a
Age, years (SD)	64.1 (15.9)	34.58 (16.1)	<0.001
Women	64 (51.6)	18 (58.1)	0.658
Ethnicity			0.023
Hispanic or Latino	11 (8.9)	8 (25.8)	
Race			0.004
Black or African American	45 (36.9)	10 (32.3)	
White	62 (50.8)	14 (45.2)	
Asian	5 (4.1)	0	
Other	5 (4.1)	7 (22.6)	
Unknown	7 (5.6)	0	
Medical history and vascular risk factors			
Hypertension	91 (73.4)	6 (19.4)	<0.001
Hyperlipidemia	69 (55.6)	5 (16.1)	<0.001
Obesity	36 (29.0)	3 (9.7)	0.035
Diabetes	45 (36.3)	3 (9.7)	0.004
Tobacco use	40 (32.3)	6 (19.4)	0.191
History of stroke or TIA	39 (31.5)	1 (3.2)	0.001
Atrial fibrillation	14 (11.3)	2 (6.5)	0.741
Valvular heart disease	15 (12.1)	0 (0.0)	0.043
Myocardial infarction	20 (16.1)	0 (0.0)	0.014
Coronary artery disease	29 (23.4)	0 (0.0)	0.001
Systolic heart failure	16 (12.9)	0 (0.0)	0.043
Family history of stroke or TIA	17 (13.7)	0 (0.0)	0.025
Malignancy	29 (23.4)	6 (19.4)	0.811
>1 Cardiovascular risk factor	113 (91.1)	14 (45.2)	<0.001
Medications on admission			
Antiplatelet	56 (45.2)	3 (9.7)	<0.001
Anticoagulation	9 (7.3)	1 (3.2)	0.688
Statin	59 (47.6)	2 (6.5)	<0.001
Antidepressant	13 (10.5)	3 (9.7)	1
Antiseizure drug	12 (9.7)	6 (19.4)	0.205
Antipsychotic	3 (2.4)	0 (0.0)	1
Chemotherapy	9 (7.3)	5 (16.1)	0.157
Antibiotic	12 (9.7)	6 (19.4)	0.205

**Table 2:** Comparison of clinical and radiological characteristics between patients with vascular and CLOCC etiologies for diffusion restriction in the splenium of the corpus callosum.

	Vascular	CLOCC	*p* Value
	N (%)	N (%)	
Clinical data			
Elevated LDL (>100)	30 (24.2)	2 (6.5)	0.027
Elevated A1c (>5.7)	70 (56.5)	5 (16.1)	<0.001
EKG findings			
ST elevation	3 (2.4)	1 (3.2)	1
ST depression	4 (3.2)	1 (3.2)	1
T wave inversion	19 (15.3)	2 (6.5)	0.252
Prolonged QT interval	10 (8.1)	2 (6.5)	1
Atrial fibrillation	9 (7.3)	1 (3.2)	0.688
EKG abnormality count ^†^			0.865
0	88 (71.0)	25 (80.6)	
1	29 (23.4)	5 (16.1)	
2	5 (4.0)	1 (3.2)	
3+	2 (1.6)	0	
Echocardiogram findings			
Intracardiac thrombus	7 (5.6)	0 (0.0)	0.346
Endocarditis	12 (9.7)	0 (0.0)	0.126
Intracardiac tumor	2 (1.6)	0 (0.0)	1
Dilated left atrium	45 (36.3)	2 (6.5)	0.001
Patent foramen ovale	17 (13.7)	1 (3.2)	0.126
Reduced ejection fraction (<40%)	10 (8.1)	0 (0.0)	0.213
Echo abnormality count ^ǂ^			<0.001
0	58 (46.8)	28 (90.3)	
1	45 (36.3)	3 (9.7)	
2	16 (12.9)	0	
3+	5 (4.0)	0	
Radiological data			
Regions of corpus callosal diffusion restriction			
Splenium only	9 (7.3)	14 (45.2)	<0.001
Splenium plus other areas of the corpus callosum	19 (15.3)	10 (32.3)	0.04
Pattern of splenial involvement			
Midline	22 (17.7)	22 (71.0)	<0.001
Lateralized	102 (82.3)	8 (25.8)	<0.001
Other areas of diffusion restriction			
Multifocal	100 (80.6)	16 (51.6)	0.002
Cortical	99 (79.8)	11 (35.5)	<0.001
Other subcortical	105 (84.7)	15 (48.4)	<0.001
Other radiological findings			
T2/FLAIR cortical or subcortical lesions	108 (87.1)	24 (77.4)	0.256
IPH or IVH	10 (8.1)	10 (32.3)	0.001
Microhemorrhage	39 (31.5)	13 (41.9)	0.292
Gadolinium enhancement	20 (16.1)	4 (12.9)	0.786
Mass effect	18 (14.5)	2 (6.5)	0.369

^†^ Sum of observed EKG findings including ST elevation, ST depression, t-wave inversion, prolonged QT interval, and atrial fibrillation. ^ǂ^ Sum of observed Echocardiogram findings including intracardiac thrombus, endocarditis, intracardiac tumor, dilated left atrium, patent foramen ovale, and reduced ejection fraction.

**Table 3:** Comparison of clinical and radiological outcomes between patients with vascular and CLOCC etiologies for diffusion restriction in the splenium of the corpus callosum.

	Frequency of Outcomes			Logistic Regression Results	
	Vascular	CLOCC	*p* Value	Odds Ratio ^†^	Adjusted * Odds Ratio
Outcomes					
Antiplatelet on discharge	66.1% (57.1%, 74.3%)	12.9% (3.6%, 29.8%)	<0.001	0.08 (0.02, 0.21) *p* < 0.001	0.15 (0.04, 0.49) *p* = 0.003
Anticoagulant on discharge	25.8% (18.4%, 34.4%)	6.5% (0.8%, 21.4%)	0.027	0.20 (0.03, 0.71) *p* = 0.033	0.60 (0.08, 2.80) *p* = 0.548
Statin on discharge	64.5% (55.4%, 72.9%)	6.5% (0.8%, 21.4%)	<0.001	0.04 (0.01, 0.13), *p* <0.001	0.12 (0.02, 0.54) *p* = 0.012
Readmission within 1 year	43.5% (34.7%, 52.7%)	32.3% (16.7%, 51.4%)	0.31	0.62 (0.26, 1.39) *p* = 0.256	1.09 (0.38, 3.14) *p* = 0.873
Resolution of diffusion restriction within 1 year	21.8% (14.9%, 30.1%)	29.0% (14.2%, 48.0%)	0.476	1.47 (0.58, 3.49) *p* = 0.394	1.0 (0.31, 3.1), *p* = 1.0
In-hospital mortality	12.1% (6.9%, 19.2%)	0%	0.043	0 (-Inf, Inf)	0 (-Inf, Inf)

^†^ CLOCC/Vascular. * Adjustment for age, sex, race (white/non-white), and ethnicity.

## Data Availability

The data are not publicly available because study participants did not consent for such availability.
